# The New Approach to the Preparation of Polyacrylamide-Based Hydrogels: Initiation of Polymerization of Acrylamide with 1,3-Dimethylimidazolium (Phosphonooxy-)Oligosulphanide under Drying Aqueous Solutions

**DOI:** 10.3390/polym13111806

**Published:** 2021-05-30

**Authors:** Natalia Tarasova, Alexey Zanin, Efrem Krivoborodov, Ilya Toropygin, Ekaterina Pascal, Yaroslav Mezhuev

**Affiliations:** 1 Institute of Chemistry and Problems of Sustainable Development, Dmitry Mendeleev University of Chemical Technology of Russia, 12047 Moscow, Russia; tarasnp@muctr.ru (N.T.); iEfrem@muctr.ru (E.K.); 201221@muctr.ru (E.P.); yamezhuev@muctr.ru (Y.M.); 2 Institute of Geology of Ore Deposits, Petrography, Mineralogy, and Geochemistry, Russian Academy of Sciences, 119017 Moscow, Russia; 3 V.N. Orekhovich Research Institute of Biomedical Chemistry, Russian Academy of Medical Sciences, 119121 Moscow, Russia; ilya@ibmh.msk.su

**Keywords:** polyacrylamide, polymerization of acrylamide, polymerization initiator, ionic liquids, sulphur

## Abstract

The new initiator of the polymerization of acrylamide, leading to the formation of crosslinked polyacrylamide, was discovered. The structure of the synthesized polyacrylamide was characterized by XRD, ^1^H NMR, and ^13^C NMR spectroscopy. It was shown that 1,3-dimethylimidazolium (phosphonooxy-)oligosulphanide is able to initiate radical polymerization under drying aqueous solutions of acrylamide, even at room temperature. According to XRF data, the synthesized polyacrylamide gel contains 0.28 wt% of sulphur. The formed polymer network has a low crosslinking density and a high equilibrium degree of swelling. The swelling rate of polyacrylamide gel in water corresponds to the first order kinetic equation with the rate constant 6.2 × 10^−2^ min^−1^. The initiator is promising for combining acrylamide polymerization with the processes of gel molding and drying.

## 1. Introduction

Linear and crosslinked polyacrylamide, as well as acrylamide copolymers, are of significant applied interest and are used in water treatment [1,2,3], enhanced oil recovery (EOR) technologies [3,4,5,6], stabilization of nanoparticles [7,8,9], electrophoresis-induced separation and purification of substances [10], and adsorption removal of Pb^2+^ ions from aqueous solutions [11]. The biocompatibility and hydrophilicity of polyacrylamide have ensured its wide application in biomedical fields [12], including in systems of pharmacologically active substance delivery [13] and the binding of toxins [14]. Several acrylamide copolymers have proven to be stimulus-sensitive polymers. For example, nanoparticles of a copolymer of acrylamide and acrylonitrile are described that are capable of transitioning from worm-like morphology to spherical when heated in water [15].

**Scheme 1:**
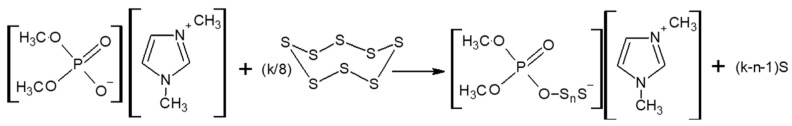
Synthesis of 1,3-dimethylimidazolium (phosphonooxy-)oligosulphanide.

For biomedical applications, polymer networks based on crosslinked polyacrylamide, capable of large, limited swelling in water, which are obtained by radical copolymerization of acrylamide and methylene-bis-acrylamide, are of particular importance. Although polyacrylamide gels are widely used, significant difficulties arise in the need for polymerization molding of articles. Therefore, the development of a method that provides the initiation of acrylamide polymerization under the conditions of drying its aqueous solutions is an important applied problem. Although the kinetics and the mechanism of polyacrylamide synthesis have been thoroughly studied [16,17], the search for new methods of initiating the polymerization of acrylamide in mild conditions continues [18,19,20]. Earlier, we discovered the possibility of ring opening of cyclooctasulphur in the presence of 1,3-dimethylimidazolium dimethyl phosphate with the formation of phosphorylated linear sulphur oligomers (Scheme 1) [21].

This work shows for the first time the possibility of using 1,3-dimethylimidazolium (phosphonooxy-)oligosulphanide to initiate polymerization of acrylamide under conditions of prolonged drying of its aqueous solution at room temperature. As a result, the formation of a crosslinked polyacrylamide was observed in the absence of methylene-bis-acrylamide.

## 2. Materials and Methods

### 2.1. Synthesis of Polyacrylamide in the Presence of 1,3-Dimethylimidazolium (Phosphonooxy-)Oligosulphanide

0.2 mL of a solution of 1,3-dimethylimidazolium (phosphonooxy-)oligosulphanide, obtained according to the previously described method [21], was added to a solution of 0.5 g of acrylamide (“Sigma-Aldrich”, purity over 99%, St. Louis, MI, USA) in 2 mL of water (bidistillate), followed by stirring for 20 min. Then the reaction mixture was left at a temperature of 298 K (25 °C) and dried. The structure of the obtained polyacrylamide was characterized by XRD (Benchtop X-ray Diffractometer, Malvern Panalytical Aeris XRD) and X-ray fluorescent analysis (Bruker Kappa APEX DUO).

The dynamics of acrylamide polymerization was investigated in situ in an open ampoule, daily recording the ^1^H NMR spectrum (Bruker CXP 200) of the reaction system. For the sample isolated four days after the start of the reaction and washing with methanol (in the calculation of 10 mL of CH_3_OH (“Sigma-Aldrich”, purity over 99%) per 1 g of gel), the ^13^C NMR spectrum was recorded.

### 2.2. Swelling Kinetics of Polyacrylamide in Water

0.115 g of synthesized polyacrylamide, thoroughly washed with methanol and dried, was placed in 100 mL of water. The system was thermostated at 298 K (25 °C), and the mass of the sample was recorded at specified time intervals.

## 3. Results and Discussion

**Figure 1:**
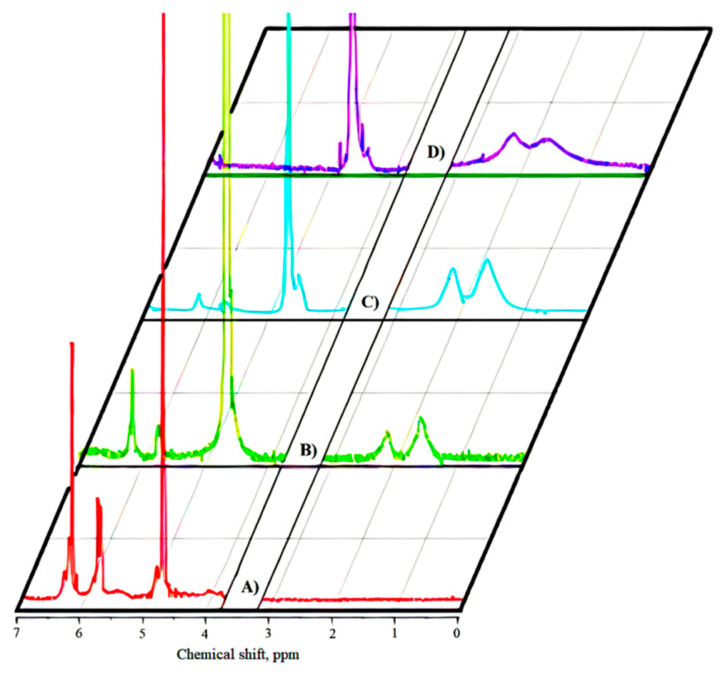
The ^1^H NMR spectra of the reaction system obtained in the process of drying an aqueous solution of acrylamide at a temperature of 298 K (25 °C) after: (**A**) 1 day; (**B**) 4 days; (**C**) 5 days; (**D**) 6 days after the start of the reaction.

The ^1^H NMR spectrum contains signals of 5.69 ppm and 6.10 ppm related to the vinyl moiety of the residual acrylamide (Figure 1A), as well as signals of 2.07 ppm and 1.52 ppm, belonging to the CH and CH_2_ groups of the polyacrylamide chain (Figure 1D). The assignment of signals on the ^13^C NMR and ^1^H NMR spectra of polyacrylamide gels are consistent with the previously described outcomes [22,23]. The ^1^H NMR spectra of the reaction system, obtained during the drying of an aqueous solution of acrylamide in the presence of 1,3-dimethylimidazolium (phosphonooxy-)oligosulphanide, indicate the gradual formation of polyacrylamide (Figure 1). The reaction proceeds almost until the complete conversion of the monomer, so that after 6 days no vinyl proton signals of the initial acrylamide were observed in the ^1^H NMR spectrum of the reaction system (Figure 1D).

**Figure 2:**
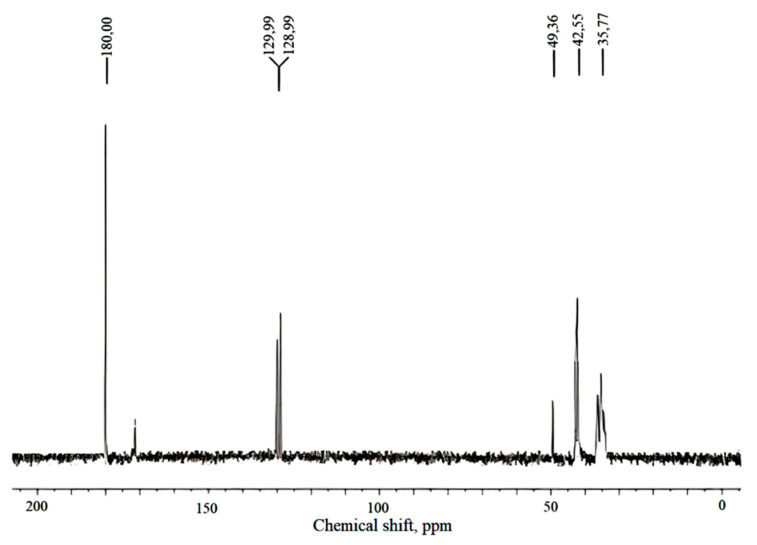
^13^C NMR spectrum of polyacrylamide gel after rinsing with methanol at the rate of 10 mL of CH_3_OH per 1 g of crosslinked polymer.

It is noteworthy that polymerization begins only when most of the water has been removed and does not develop in a sealed ampoule. The polymerization of acrylamide in the presence of 1,3-dimethylimidazolium (phosphonooxy-)oligosulphanide is evidenced by the ^13^C NMR spectrum recorded in D_2_O for gel washed with methanol (Figure 2). As observed, the ^13^C NMR spectrum contains signals of 171 ppm, 129.99 ppm and 128.99 ppm related to the carbon atoms of residual acrylamide, as well as signals of 180.00 ppm, 42.25 ppm and 35.77 ppm, related to the carbon atoms of the C=O, CH and CH_2_ moieties of the resulting polyacrylamide. The signal with a chemical shift of 50 ppm refers to the methanol carbon used for rinsing.

**Figure 3:**
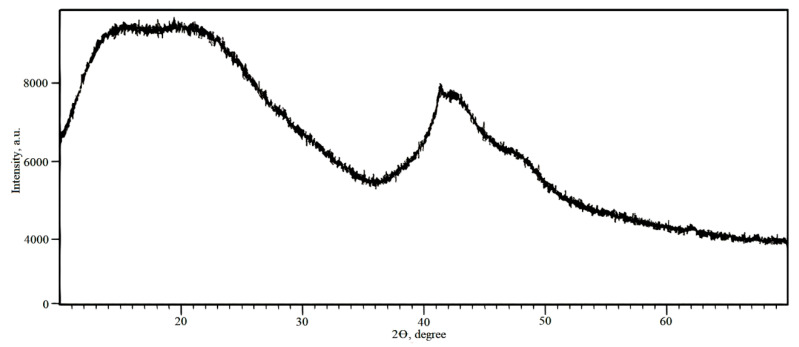
XRD of dried polyacrylamide gel.

XRD data indicate the formation of an amorphous substance, which is characteristic of polyacrylamide (Figure 3) [24]. According to X-ray fluorescent analysis, the mass proportion of sulphur within the polyacrylamide gel sample repeatedly purified by rinsing with methanol and distilled water is 0.28 wt%, while the ^31^P NMR spectrum recorded by using methanol for rinsing shows a singlet with a chemical shift of 3.28 ppm (relative to 85% aqueous phosphoric acid), which is characteristic for the phosphorus atom of the dimethyl phosphate anion.

**Figure 4:**
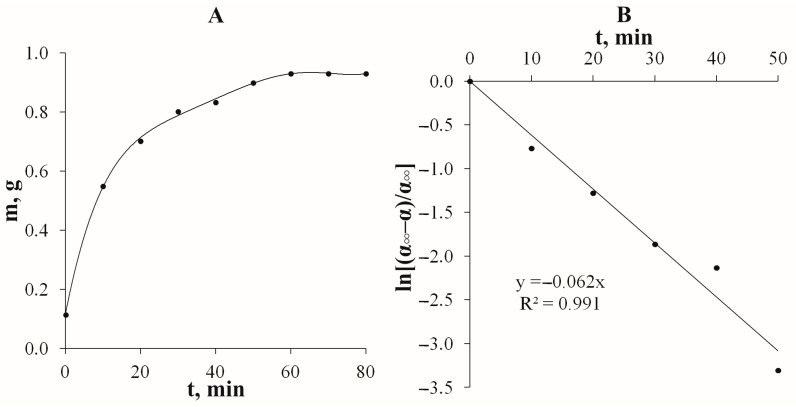
Kinetic curves of the swelling of polyacrylamide in water at a temperature of 298 K (25 °C) in coordinates: (**A**)—m vs. t; (**B**)—$\ln\left[\frac{\left(\alpha_{\infty }-\alpha \right)}{\alpha_{\infty }}\right]$ vs. t, where m—current value of gel weight.

The synthesized polyacrylamide was only capable of limited swelling in water, which indicates the formation of a three-dimensional polymer network (Figure 4). The linearity of the kinetic curve of the swelling of polyacrylamide in water in coordinates $\ln\left[\frac{\left(\alpha_{\infty }-\alpha \right)}{\alpha_{\infty }}\right]$ vs. t indicates the subordination of the process to the quasi-first order Equation (1) and its integral form (2):

(1) $$\frac{d\alpha }{dt}=k\left(\alpha_{\infty }-\alpha \right)$$

(2) $$\ln\left[\frac{\left(\alpha_{\infty }-\alpha \right)}{\alpha_{\infty }}\right]=-kt$$

where: $\alpha =\left(m-m_{0}\right)/m_{0}$—the degree of swelling and $\alpha_{\infty }$—the equilibrium degree of swelling of the polyacrylamide gel in water; $m_{0},\;m$—initial and current value of gel weight; t—time; k—swelling rate constant.

The swelling rate constant of polyacrylamide in water was 6.2 × 10^−2^ min^−1^, and the equilibrium degree of swelling is 7.09. The obtained kinetic data make it possible to quantitatively estimate the average molecular weight of the chain segments between the network nodes according to the Flory and Rehner Equation (3) [25]:

(3) $${\bar{M}}_{c}=-\frac{d_{P}V_{mS}\left(\varphi^{\frac{1}{3}}-0.5\varphi \right)}{\ln\left(1-\varphi \right)+\varphi +\chi \varphi^{2}}$$

where: ${\bar{M}}_{c}$—average molecular weight of chain segments between network nodes; φ—volume fraction of the polymer that has reached equilibrium swelling; $d_{P}$—density of the polymer; $V_{mS}$—molar volume of the solvent; χ—Huggins interaction parameter.

Taking into account the density of polyacrylamide 1.3 g × cm^−3^, the volume fraction of the polymer (φ) upon reaching the equilibrium degree of swelling is 0.098. Since the Huggins interaction parameter for the polyacrylamide—water system is close to 0.493 at a temperature of 298 K (25 ℃) [26], ${\bar{M}}_{c}$ is about 10^4^. Therefore, the use of 1,3-dimethylimidazolium (phosphonooxy-)oligosulphanide as an initiator of acrylamide polymerization under conditions of slow drying of its aqueous solutions allows the formation of a lightly crosslinked polymer network with high water absorption.

**Scheme 2:**
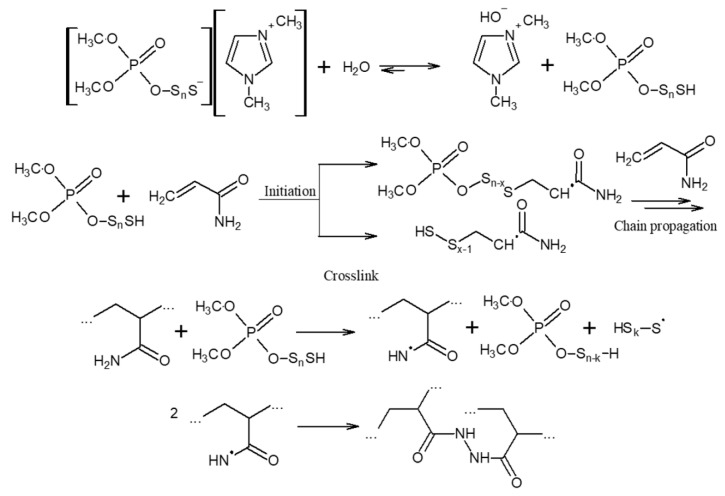
The proposed mechanism for the formation of crosslinked polyacrylamide in the presence of 1,3-dimethylimidazolium (phosphonooxy-)oligosulphanide.

The listed circumstances can be explained if we admit the mechanism shown in Scheme 2. In accordance with this mechanism, the initiation of polymerization is possible only under the condition of a high concentration of acrylamide in the reaction system. In addition, according to the HSAB theory, only a low rate of processes leading to chain crosslinking can be expected, which is consistent with the formation of polymer gels with a high equilibrium degree of swelling and low crosslinking density.

The possibility of initiating polymerization of acrylamide with 1,3-dimethylimidazolium (phosphonooxy-)oligosulphanide is consistent with the increased ability for the formation of S–S bonds to homolytic decomposition, compared to S_8_ [27,28,29,30,31] and the low stability of hydrogen polysulphides. Although the details of the mechanism of the reaction described above have yet to be established, we report on the fundamental possibility of initiating radical polymerization of acrylamide with 1,3-dimethylimidazolium (phosphonooxy-) oligosulphanide.

## 4. Conclusions

The product of the interaction of elemental sulphur with 1,3-dimethylimidazolium dimethylphosphate is capable of initiating acrylamide polymerization upon the slow evaporation of water at room temperature. The resulting polyacrylamide is capable of limited swelling in water, and the equilibrium mass degree of swelling is 7.09, and the swelling rate constant at a temperature of 298 K (25 °C) is 6.2 × 10^−2^ min^−1^. Thus, simultaneously with the formation of polyacrylamide chains, the process responsible for their crosslinking develops, which opens the possibility of obtaining polymer networks by drying aqueous solutions of acrylamide. This reaction opens new perspectives and modes of forming articles from crosslinked polyacrylamide.

Apparently, both the initiation of polymerization of acrylamide and the crosslinking of the chains of the resulting polyacrylamide are a consequence of the low stability of the S–S bonds of linear sulphur chains of 1,3-dimethylimidazolium (phosphonooxy-)oligosulphanide.

## Data Availability

Not applicable.
